# Xiaochaihu decoction for nonalcoholic fatty liver disease: A protocol for a systematic review and meta-analysis

**DOI:** 10.1097/MD.0000000000025267

**Published:** 2021-03-26

**Authors:** Wenyu Zhu, Huan Luo, Min Xiong, Tao Shen

**Affiliations:** College of basic medicine, Chengdu university of Traditional Chinese Medicine, Chengdu, Sichuan, China.

**Keywords:** non-alcoholic fatty liver disease, protocol, systematic review, Xiaochaihu decoction

## Abstract

**Background:**

Nonalcoholic fatty liver disease (NAFLD) is a clinicopathologic syndrome characterized by excessive deposition of fat in hepatocytes except for alcohol and other specific hepatic factors. Xiaochaihu decoction (XD) has been widely used to treat NAFLD in China. However, there is no systematic review found. In order to evaluate the efficacy and safety of XD in the treatment of NAFLS, we need to conduct a meta-analysis and systematic evaluation.

**Methods:**

There are enrolled randomized controlled trials (RCTs) evaluating the effectiveness and safety of XD in the treatment of NAFLD. Data come mainly from 4 Chinese databases (CNKI, CBM, Wanfang, and VIP Database) and 4 English databases (Pubmed, Embase, Cochrane Library, and Web of science). The enrollment of RCTs is from the starting date of database establishment till September 30, 2021. The work such as selection of literature, data collection, quality evaluation of included literature, and assessment of publication bias will be conducted by 2 independent researchers. Meta-analysis will be performed by RevMan 5.0 software.

**Results:**

This study will provide high-quality evidence for the effectiveness and safety of XD in the treatment of NAFLD.

**Conclusion:**

The results of the study will help us determine whether XD can effectively treat NAFLD.

**Ethics and dissemination:**

This study does not require ethical approval. We will disseminate our findings by publishing results in a peer-reviewed journal.

**OSF registration number:**

DOI 10.17605/OSF.IO/A5XEM

## 1 Introduction

Nonalcoholic fatty liver disease (NAFLD) is defined as the accumulation of excessive fat in more than 5% of hepatocytes without significant alcohol intake.^[1]^ NAFLD is a progressive liver metabolic disease with a broad spectrum of histological abnormalities, ranging from simple steatosis to nonalcoholic steatohepatitis, cirrhosis, and even hepatocellular carcinoma.^[2,3]^ NAFLD, especially nonalcoholic steatohepatitis, is often associated with type 2 diabetes mellitus, NAFLD, and central obesity.^[4]^ Liver diseases that progress to fibrosis and cirrhosis are irreversible, and the only option for treatment is liver transplantation, which is a substantial economic burden worldwide.^[5]^ The liver is an essential metabolic organ that principally impacts fatty acid β-oxidation, lipid catabolism, and other physiological processes, such as glucose homeostasis and adipocyte differentiation.^[6]^ In the setting of overnutrition, hepatic fatty acid metabolism is altered, the excessive triglyceride within hepatocytes aggravates chronic inflammatory response, and metabolic disturbance.^[7,8]^ It is predicted that in the next 10 years, more than half of the world's population will be at risk of NAFLD.

At present, the treatment of NAFLD is mainly based on the patient's own lifestyle and dietary adjustments. Available drugs include vitamin E, pioglitazone, and obeticholic acid, but there is no targeted therapy. Therefore, finding and exploring new treatment strategies and targets, and intervening in the occurrence and development of NAFLD have important clinical significance and social value. The superiority and therapeutic effects of traditional Chinese medicine on NAFLD have been gradually recognized and developed in the course of clinical practice. Xiaochaihu decoction (XD), a compound recipe composed of Chaihu, Huangqin, Banxia, Shengjiang, Renshen, Dazao, and Gancao, has been confirmed effective in treating NAFLD. In this article, we aim to perform a systematic review to evaluate effectiveness and safety of XD in the treatment of NAFLD to provide reference for clinical application.

## 2 Methods

### 2.1 Protocol and registration

The protocol has been registered on the Open Science Framework (OSF) platform (https://osf.io/tahnd/), registration number: DOI 10.17605/OSF.IO/A5XEM. This protocol was drafted and reported in accordance with the Preferred Reporting Items for Systematic Reviews and Meta-Analyses Protocols (PRISMA-P) guidelines.^[9]^ The final report will comply with the recommendations of the PRISMA Extension Statement for Reporting of Systematic Reviews Incorporating Meta-analyses of Healthcare Interventions.^[10]^

### 2.2 Ethics

We will not need individual data of each patient in the research as this is a systematic review. Therefore, institutional review board approval and ethics committee is not needed. Our purpose is to publish the results in a peer-reviewed journal. The final results of the review will provide information about the safety and efficacy of XD and its modified forms in the treatment of NAFLD to help clinicians make decisions on clinical practice.

### 2.3 Eligibility criteria

The participant (P), intervention (I), comparator (C), outcome (O), and study design (S) are the 5 main factors determining the inclusion and exclusion criteria of this research.

#### 2.3.1 Type of study design

We will exclude quasi-RCT, non-RCT, observation group combination other drugs, animal experiments, control groups not match, and full texts not available by reading the title, abstract and related quotations Information screening. Then we can eliminate incomplete experimental data and experimental design schemes not rigorous, no clear diagnostic criteria by reading and understanding the full article, and finally randomized controlled trials were included this research.

#### 2.3.2 Type of participant

Patients who have been diagnosed as NAFLD will be included. There are no restrictions on sex and age. Patients combined with other chronic liver diseases such as alcoholic liver disease, various viral hepatitis, autoimmune liver disease, drug-induced hepatitis will be excluded. Additionally, liver cirrhosis, liver cancer, severe liver disease requiring liver transplantation, and other complicated and severe patients are also not included.

#### 2.3.3 Type of interventions

The patients in the treatment groups will be given XD or modified XD as a monotherapy or in combination with conventional therapy. XD consists of 7 herbs: Chaihu, Huangqin, Banxia, Shengjiang, Renshen, Dazao, and Gancao. The number of modified herbs will not exceed 3 (n ≤ 3). Patients of control group will be treated with conventional therapy with placebo, stains and fibrates. In addition, the 2 groups have not taken any drugs that possibly interfered with the outcome indicators. The follow-up time was ≥4 weeks.

#### 2.3.4 Type of outcomes

1. Primary outcomes: Total effective rate, main blood lipid indexes (TC, TG), main liver function indexes (ALT, AST).
2. Secondary outcomes: TCM Syndrome Score Scale (TCMSSS), body mass index (BMI), fasting blood glucose, ultrasound imaging changes, and the incidence of adverse events, etc.
3. Safety outcomes: Safety indicators consist of liver enzyme, kidney function and fasting blood glucose.

### 2.4 Literature retrieval strategy

Computer search included PubMed, the Cochrane Library, EMbase, China National Knowledge Infrastructure (CNKI), China Biomedicine (CBM), Chinese Scientific Journals Database (VIP), Wanfang Database for published information about RCTs of XD for the treatment of NAFLD. The time limit for literature search is from the establishment of each database to September 30, 2020. The language is limited to English and Chinese. In addition, the World Health Organization International Clinical Trials Registration Platform and Clinical Trials website (Clinical Trials.gov) will be searched for ongoing trials related to the disease and RCTs in China. The search method uses a combination of free words and medical subject terms, including: “non-alcoholic fatty liver disease,” “Chinese medicine,” “Xiaochaihu decoction,” among others. Chinese database search will use the following terms: “zhifanggan,” “feijiujingxing,” “gandan,” “zhongyao,” “xiaochaihutang,” among others. Taking PubMed as an example, the initial search strategy is shown in Table 1, which will be adjusted according to the specific database.

**Table 1:** Search strategy of the PubMed.

No.	Search terms
#1	Non-alcoholic fatty liver disease [Mesh]
#2	Non-alcoholic fatty liver disease [Title/Abstract] OR NAFLD [Title/Abstract] OR Liver disease [Title/Abstract]
#3	#1 OR #2
#4	Xiaochaihu [Title/Abstract]
#5	Decoction[Title/Abstract]
#6	#4 AND #5
#7	randomized controlled trial[Publication Type]
#8	controlled clinical trial[Publication Type]
#9	randomized[Title/Abstract]
#10	randomly[Title/Abstract]
#11	#10 OR #11 OR #12 OR #13
#12	#3 AND #6 AND #11

### 2.5 Data collection and analysis

#### 2.5.1 Literature selection and data extraction

**Figure 1:**
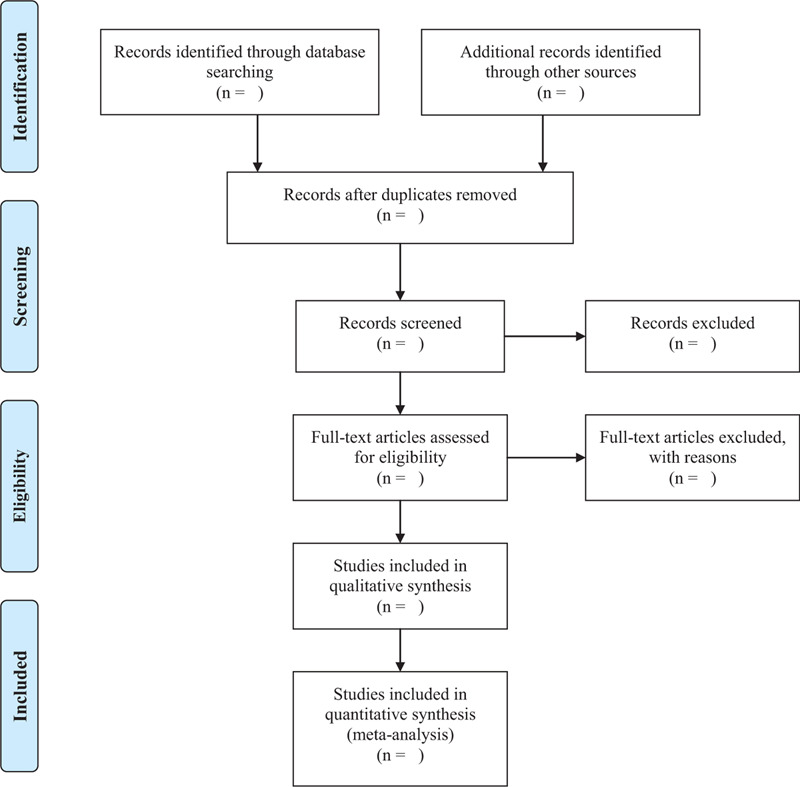
PRISMA flow diagram of the study selection process.

As shown in Figure 1, 2 researchers (WZ and MX) will screen the documents according to the inclusion and exclusion criteria: import the retrieved documents into Endnote X9 software for review, then remove duplicate references; by preliminary screening abstract, we exclude documents which obviously do not meet the inclusion criteria; download and read the full paper for follow-up examination; after the final inclusion, we will use the predesigned data extraction table for data extraction and cross-check the results; if there is any objection, the third researcher (HL) will be asked to assist in the judgment. The main content of data extraction includes: basic information of literature (title, journal, author, publication date), basic information of the research object (sample size, sex, average age, intervention, and course of treatment), and result data (numbers of response events, non-response events, dropouts, time points, mean, SD, follow-up time and adverse events). If the required information is missing or incomplete, we will contact the relevant email address of the corresponding author or first author of the original document. If the relevant data cannot be obtained, the record is excluded. At the same time, the key factors of bias risk assessment are extracted.

#### 2.5.2 Risk of bias assessment

The method quality of systematic review reflects the risk of deviation or validity in its process and results. The quality of the method will be evaluated according to Cochrane Manual 5.2.0.^[11]^ Two well-trained researchers (WZ and MX) independently assessed the risk of bias in the study. The evaluation content includes generation of random sequences, randomization concealment, the implementation of blinded subjects and researchers, the implementation of blind methods for outcome evaluators, the integrity of outcome data, selective reporting, and other biases. Each item should be judged as 3 “low risk” levels bias, “high-risk bias,” and “unclear” follow quality classification standards. For each item, if it is satisfactory, it means “low risk deviation,” if not, it means “high risk deviation.” When there is not enough information in the literature to make a clear judgment on the corresponding item, it means “unclear.” If there is any dispute, we will submit it to the corresponding author (TS) for arbitration.

#### 2.5.3 Data synthesis

Meta-analysis will be performed by RevMan 5.0 software (Version 5.3, Copenhagen: The Nordic Cochrane Center, 2014) provided by the Cochrane Collaboration. Although there is statistical homogeneity between each study (*I*^2^ < 50%), the fixed-effect model is used. When the heterogeneity is significant (*I*^2^ ≥ 50%) between the results of each study, the sublayer analysis is performed to find the source of heterogeneity. A fixed effect model is used for meta-analysis when there is sufficient similarity between the results of the subgroups (*I*^2^ < 50%). However, a random-effect model is used for meta-analysis if the heterogeneity between the results of the subgroups is significant (*I*^2^ ≥ 50%). Qualitative heterogeneity is used when heterogeneity is too large or the source of heterogeneity is unknown. Meta-regression analysis can be performed if there are many influencing factors and it is not appropriate to use the stratification method.

#### 2.5.4 Assessment of heterogeneity

Before the combination of effect size, the heterogeneity of the included literature is tested using Stata 13.0. When interstudy heterogeneity exists, the random effect model will be used. For comparison of each pair, Heterogeneity is determined by heterogeneity test and expressed by *I*^2^ value. When *I*^2^ ≥ 50%, the heterogeneity is large. When 25% < *I*^2^ < 50%, we think it is moderate. When *I*^2^ < 25%, the heterogeneity is considered small.

#### 2.5.5 Sensitivity analysis

Sensitivity analysis is based on sample size, missing data results, and methodological quality. Sensitivity analysis will be put into effect to examine the robustness of the pooled results in case of sufficient data by determining the effects of excluding studies with high risks of bias or with missing data, and outliers.

#### 2.5.6 subgroup analysis

When *I*^2^ > 50% and P value < .1, there is obvious heterogeneity between the studies, therefore it is essential to analyze the reasons for that. We will conduct a subgroup analysis according to the source of heterogeneity. For instance, if it is due to the quality of methodology, subgroup analysis will be undertaken in the light of the quality. If it is due to the difference in design, we will analyze age, gender, treatment type, and course of disease.

#### 2.5.7 Assessment of reporting bias

If ≥10 articles are conducted, a comparison-adjusted funnel plot is developed to using Stata to evaluate the presence of small sample effects or publication bias in the intervention. Descriptive analysis will be carried out by the method of the symmetry of funnel plot. If there is asymmetric or no inverted funnel in the plot, it is deemed that there may be publication bias. It is possibly connected with the difficulty in the publication of the literature with negative results and the low quality of the inclusion methods.

#### 2.5.8 Grading the quality of the evidence

To grade evidence quality and understand the actual situation of evidence rating thereby analyzing possible questions, the Grading of Recommendations Assessment, Development and Evaluation (GRADE) system will be used to evaluate the quality of evidence.^[12]^ On account of the risk of bias, imprecision, inconsistency, indirection, and publication bias, GRADE grades evidence quality into 4 levels: high, medium, low, and very low.

## 3 Discussions

NAFLD is a chronic progressive liver disease with a continuum of harmful conditions that disturb metabolic processes.^[13]^ As an excessive supply of nutrients causes NAFLD, promising lifestyle modifications, such as weight loss through exercise can alleviate NAFLD. However, it remains challenging to achieve and sustain such modifications for most patients, and a substantial proportion of them are dramatically lean.^[14,15]^ Many studies have demonstrated XD's efficacy as a hypolipidemic and NAFLD protectant in animal experiments and clinical trials, making it a candidate for future therapy against NAFLD. Otherwise, there is no systematic review and meta-analysis to evaluate its efficacy and safety at present. Therefore, it is necessary to provide compelling evidence for the advantages of XD in NAFLD through a high-quality systematic review and meta-analysis. In addition, there may still be potential shortcomings in this study. First of all, the form of research in Chinese and English will likely increase the bias of research. Secondly, age, gender, race, drug formulation, dosage, and course of treatment can lead to the risk of heterogeneity. Finally, the study may involve a small number of clinical trials, leading to a high risk of bias.

## Author contributions

Wenyu Zhu and Min Xiong made the same contribution to the research and design, and wrote the original draft of the protocol. Wenyu Zhu has developed a search strategy. Wenyu Zhu, Min Xiong and Huan Luo will conduct literature retrieval and collation. Wenyu Zhu, Min Xiong and Tao Shen will evaluate the risk of bias in the literature. Data analysis and article writing will be done by Wenyu Zhu, Min Xiong. Tao Shen, as the corresponding author, will be responsible for overseeing every process of the audit review to control the quality of the study. All the authors have approved the publication of the protocol.

**Conceptualization:** Wenyu Zhu.

**Data curation:** Huan Luo.

**Funding acquisition:** Tao Shen.

**Investigation:** Huan Luo.

**Methodology:** Wenyu Zhu.

**Project administration:** Tao Shen.

**Validation:** Min Xiong.

**Visualization:** Min Xiong.

**Writing – original draft:** Wenyu Zhu.
